# How the availability and adequacy of social support affect the general mental health of Swedish police officers

**DOI:** 10.3389/fpsyg.2023.1196320

**Published:** 2023-10-16

**Authors:** Jonas Hansson, Mojgan Padyab

**Affiliations:** ^1^Police Education Unit, Umeå University, Umeå, Sweden; ^2^Department of Social Work, Umeå University, Umeå, Sweden; ^3^Center for Demographic and Aging Research (CEDAR), Umeå University, Umeå, Sweden

**Keywords:** emotional support, mental health, police, social interaction, social support, Sweden

## Abstract

Police work is stressful. A protective function against work stress and harm to mental health is social support, either within or outside work. This cross-sectional study analyzes the associations of quantitative (availability) and qualitative (adequacy) aspects of social support with general mental health among Swedish police officers. A total of 728 officers responded to a national survey. Bivariate analyses (*t*-test and chi square) identified continuous and categorical variables (respectively) statistically significantly associated with sex and social support. Pearson correlation coefficient was provided to indicate the associations between general mental health and different types of social support. Sex-stratified logistic regression modeling calculated crude and adjusted odds ratios (OR) with 95% confidence intervals (CI) and assessed the relationships between different types of social support, sociodemographic variables and general mental health. The findings show that low adequacy of attachment is associated with poorer mental health among female officers, although female officers also reported higher availability of both social interaction and attachment compared to male officers. We found an association between low work-related social support and poorer mental health among single male police officers. Moreover, police officers who worked shifts, were younger, had less work experience, and/or had no children reported higher availability of attachment, whereas older police officers reported higher adequacy of social interaction compared to younger police officers. Variation in the quantity and quality of close social relationships seems to be important to mental health. Police organizations need to be aware of this in their efforts to make the work environment more supportive. Social support might create an environment where officers feel more comfortable discussing their mental health concerns and seeking assistance.

## 1. Introduction

“It’s hard to afford to have a bad day. It can be about having slept badly or having a hard time in private. You should then be able to talk about it at the roll call and be relieved. But in reality, if all patrols are busy and there is a traffic accident with serious injuries or if someone jumped in front of the train, then we have to deal with it anyway.” (Group interviews with patrolling police officers in Stockholm, February 2020)

Patrolling police officers must be able to build trust by being authoritative but also relational. This places high demands on their mental balance: in the same location, they may be pelted by stones one day then spend the next trying to build relationships. Police work is inherently stressful (Stinchcomb, 2004; Maguen et al., 2009; Chopko, 2010; Garbarino et al., 2011; Backteman-Erlanson et al., 2013; Ménard and Arter, 2013; Russell, 2014; Padyab et al., 2016; Gutschmidt and Vera, 2021; Nourry et al., 2023). However, the fellowship within the police profession, besides being a strong factor for job satisfaction, also fulfills the function of helping officers cope with demanding and stressful work (Sundqvist et al., 2021). This fellowship provides social support, which has been found to protect against work stress and mental health, either within or outside work (Cohen and Wills, 1985; Viswesvaran et al., 1999; Michie and Williams, 2003; Stansfeld and Candy, 2006). Social support has also been found to be associated with resilience (Bonanno et al., 2007). Its protective function has been identified in studies of police officers (Marmar et al., 2006; Morash et al., 2006; Prati and Pietrantoni, 2010; Schwarzer et al., 2013; Arble et al., 2018; Sherwood et al., 2019; Syed et al., 2020). Prior research has investigated general mental health, job demands and control, and social support within and outside work for Swedish police and social workers involved in deporting unaccompanied, asylum-seeking refugee children (Sundqvist et al., 2015; Hansson et al., 2017; Sundqvist et al., 2017). In one of these studies, low levels of satisfaction with close emotional support and low levels of satisfaction with social interaction were related to experience of psychological disturbances (Sundqvist et al., 2017). Another study showed that the joint effect of high job strain and low work-related social support were associated with poor mental health (Hansson et al., 2017). However, work-related social support, and social support, consisting of both quantitative (availability) and qualitative (adequacy) aspects of social interaction and emotional support, and general mental health in relation to different functions of police work are meager.

Although definitions vary widely, social support is a crucial part of relationships (Virtanen and Isotalus, 2012). Social support can be defined as the emotional or practical assistance that a person receives from social groups, such as family, friends, and colleagues, during times of crisis and distress (Henderson et al., 1980). These social groups provide the opportunity to augment one’s quality of life and protect oneself against adverse life events (Prati and Pietrantoni, 2010; Setti et al., 2016; Guilaran et al., 2018). Social support sources (i.e., family, friends, and colleagues) can be referred to as structural support, and the actual support they provide to an individual can be referred to as functional support (House et al., 1985). In addition, functional support can be divided into four dimensions: instrumental (material or physical help), informational (information during times of crisis), appraisal (relevant information for self-evaluation, rather than problem solving), and emotional support (care, empathy, love, and trust) (Heaney and Israel, 2008; Muñoz-Laboy et al., 2014). Social support can also be conceptualized as enacted social support, the actual social resources provided to an individual (Barrera, 1986), and perceived social support, the belief that help is available if needed (Sarason et al., 1991). This study focuses on both structural and functional support, i.e., the “availability of persons or the provisions supplied by them” and “the perceived adequacy of this for the respondent” (Henderson et al., 1980, p. 725). It is plausible, for instance, for a person to have a high level of availability but a low level of adequacy in social relationships (Henderson et al., 1980). Studies have shown that social support varies as people age. Henderson et al. (1980) suggest that requirements of social support diminish with age. Song et al. (2023) suggest that the influence of social support on aspects like effort perseverance and interest consistency varies across different age groups, with gender playing a moderating role. The effect of social support on these matters declined as age increased. The results showed that the moderating role of age in the relationship between social support and perseverance of effort was significant among males but not females. In other words, regardless of whether females were early adolescents or late adolescents, social support consistently had a positive influence on their ability to persevere in their efforts.

A considerable amount of literature has been published on social support in relation to mental health. These studies often focus on the mediating or buffering effect of social support on different measures of mental health. Since Cohen and Wills (1985) outlined the stress-buffer hypothesis, multiple studies have proved the protective effect of social support on many different mental health disturbances. For example, lack of social support (Brewin et al., 2000) and perceived social support (Ozer et al., 2003) have been found to be robust determinants of post-traumatic stress. Further, social support might moderate the stressor-distress relationship after disasters (Arnberg et al., 2012). Social support depends on how an individual perceives support from their social network, and this directly impacts on psychological outcomes. Importantly, research has shown that social support is strongly linked to positive mental health. For example, Kshtriya et al. (2020) highlight the importance of social support for the mental health of first responders.

Various studies have investigated associations between social support and both work stress and different mental health problems among police officers. For example, a small association between social support and work stress was found in US police officers (Morash et al., 2006), while Schwarzer et al. (2013) found that social interaction buffered work stress among police officers on duty during the World Trade Center (WTC) attacks on 9/11. These differences may be influenced by how social support was conceptualized and measured in each study, as well as the unique circumstances and stressors faced by the participants. A recent study underscores the importance of social support in the context of first responders who survived the WTC attacks. It suggests that having a strong support network can enhance the effectiveness of forward-focused coping strategies, helping to reduce the severity of PTSD symptoms in these individuals (Kowalchyk et al., 2023). Another recent study found that higher social support quality was significantly related to lower endorsement of depression, anxiety, anger, and post-traumatic stress disorder (PTSD) symptoms in Canadian police officers (Nero et al., 2022). In a cross-sectional sample of 715 US police officers, lower levels of social support were associated with greater PTSD symptoms (Marmar et al., 2006). In addition, the role of workplace social support is crucial in mitigating the negative effects of work–life conflict on job dissatisfaction among police investigation officers (Shakoor et al., 2023).

Prati and Pietrantoni’s (2010) meta-analysis contributed to four main findings: (1) gender and age did not influence on the association between social support and mental health; (2) the effect size of received social support is higher compared to that of perceived social support; (3) social support is a resilience factor in the aftermath of potentially traumatic events; and (4) the mechanisms linking social support to adaptation are still unclear. In a systematic literature review, Sherwood et al. (2019) concluded that social support is strongly correlated with the psychological well-being of police officers and a key protective factor to improve their resilience. They also identified low social support from coworkers as the most common risk factor for adverse psychological outcomes. In a systematic review and meta-analysis, three independent reviewers searched 16 databases and screened 11,506 articles published between January 1980 and October 2019. Among other findings, they revealed that higher levels of peer support correlated with significantly lower PTSD symptoms (Syed et al., 2020).

A study of Swedish police officers showed that high social support was correlated with greater post-traumatic growth but not significantly related to well-being. However, police officers showed greater benefit from social support than other first responders (Arble et al., 2018). A Canadian study of public safety personnel found that perceived social support differed by occupation, with only paramedics reporting lower social support than police officers. Public safety personnel reporting greater social support were less likely to screen positive for PTSD (Vig et al., 2020). Another Canadian study reported that social support seemed to protect against anxiety-related disorders among participating police cadets. The study also found that reductions in perceived levels of social support may be a function of the Royal Canadian Mounted Police (RCMP) service since police cadets’ perceived levels of social support are comparable to the Canadian general population but higher than serving RCMP (Nisbet et al., 2023). In addition, a cross-sectional Indian study found significant positive relationship between age, social support and well-being among police personnel (Padhy et al., 2023).

Overall, these studies highlight the need for research on different types of social support and their diverse associations with different sociodemographic characteristics. Although a large literature demonstrates the protective effect of social support on mental health in police officers, there is a lack of systematic research into whether different types of social support may be more beneficial for police officers at certain life stages. Our purpose is to extend the literature on different types of social support, including availability and adequacy of social support in relation to general mental health. We have hypothesized that the mental health effects of social support depend on both availability and adequacy of social integration (social network) and attachment (emotional support). The first objective of this study was to specifically investigate availability and adequacy of aspects of different types of social support (social integration and attachment) in relation to general mental health among Swedish police officers. The second objective was to assess sex difference in term of the associations between different types of social support. The third objective was to assess the role of sociodemographic variables between different types of social support.

## 2. Method and materials

### 2.1. Setting

Until January 1, 2015, the Swedish Police Service comprised 21 police authorities (before the subsequent reorganization into the National Police Authority). These 21 authorities had geographical areas of responsibility demarcated by county boundaries. Each police authority had responsibility for daily police activities, overseen by a chief commissioner and a police board with government-appointed politicians. In general, every police authority included an investigation and legal unit, a crime prevention unit, and a service unit. In 2014, approximately 30% of the 19,911 police officers were women, and the average age of all officers was 43.8 years (National Police Board, 2014). The 21 police authorities were governed by the Swedish National Police Board, led by the National Police Commissioner.

### 2.2. Sample and data collection

All 21 police authorities in Sweden were contacted. The project was described in a letter and through conversations with one researcher’s contacts within police authorities. Three police authorities declined to participate in the study. The other 18 authorities received the survey via their human resource unit or squad leaders. A paper survey was used due to confidentiality and safety regarding use of the Internet. The surveys and prepaid return envelopes were sent in a sealed envelope to the contact person at each authority, who distributed them to police officers. The questionnaire was accompanied by an introductory letter stating the purpose of the study and a consent form. The participants were informed that the aim was to investigate how police officers experience and handle their workload, stress, and health in the context of their work with deportation of unaccompanied asylum-seeking children (UASC). The research intended to determine if there were differences in the perceived health of police officers working with deportation of UASC compared to those with other duties within the police force. Therefore, it was important to include both police officers with and without experience of UASC. In total, 728 police officers returned completed questionnaires in a prepaid return envelope to the researcher during 2014. Participants were not provided any incentive to complete the survey.

The self-administered questionnaire collected information on participants’ sociodemographic characteristics, social support inside and outside work, and general mental health. Sociodemographic characteristics included age, sex, marital status, external/internal service, children, work experience, education, and shift/daytime work. Marital status was categorized into two groups: married/cohabiting and single, with the latter including separated/divorced and widowed. Work experience referred to the number of years participants had been working in the profession. Education was a two-category variable: upper secondary education and higher education.

This study was approved by the Regional Ethical Review Board at Umeå University, Sweden, Diarie number 2014/69-31.

### 2.3. Measures

#### 2.3.1. Social support

Social support inside and outside work, including social network and emotional support, was measured by an abbreviated (30-item) Swedish version of the Interview Schedule for Social Interaction (ISSI) (Henderson et al., 1980). This instrument has previously been found to have good validity and reliability in Sweden (Undén and Orth-Gomér, 1989). The ISSI uses self-report items addressing both broader social interaction important for participation in society (social integration) and close relationships fundamental to human development (attachment). The quantity (availability) and quality (adequacy) of these dimensions are assessed through four subscales: availability of attachment, availability of social integration, adequacy of attachment, and adequacy of social integration.

Availability of social integration (AVSI) describes the magnitude of a person’s social network and contacts that help with managing everyday stress. AVSI is measured by six items, e.g., “These days, how many people with similar interests to you do you have contact with, both at work and during leisure time? How many can you openly talk to, without thinking twice?” Adding up the scores for the six items produced a value on scale from 0 to 6, with higher values indicating higher support. Availability of attachment (AVAT) describes the extent of close emotional support and appreciation within and outside the family. AVAT is measured by six items, e.g., “Is there any particular person you feel you can lean on?”; “When you are happy, is there any particular person you can share it with: someone who you feel sure will feel happy simply because you are?” Adding up the scores for the six items produced a value on a scale from 0 to 6, with higher values indicating higher support. Adequacy of social integration (ADSI) indicates qualitative aspects of a person’s social network and contacts that help with managing everyday stress. ADSI is measured by eight items, e.g., “Would you like more or less of this, or is it about right?” Adding up the scores for the eight items produced a value on a scale from 0 to 8, with higher values indicating higher support. Finally, adequacy of attachment (ADAT) indicates qualitative aspects of close emotional support and appreciation within and outside the family. ADAT is measured by ten items, e.g., “Would you like to be able to lean more or less on this person?”; “Would you like to have someone else like this or is he/she enough?” Adding up the scores for the ten items produced a value on a scale from 0 to 10, with higher values indicating higher support.

Response options for the items included: “yes” or “no”; “less,” “about right,” or “more”; or a five-point Likert scale for identifying the number of times participants have contact with different categories of people on an average day (1 = “None” to 5 = “More than 15”). Each subscale was dichotomized by mean value for men and women separately (AVSI: male 3.8 (±1.5), female 4.2 (±1.5); AVAT: male 4.1 (±1.1), female 4.4 (±1.0); ADSI: male 5.8 (±1.8), female 5.9 (±1.8); ADAT: male 7.6 (±1.8), female 7.3 (±1.9)). A value above mean indicating higher social support whereas a value below mean indicating lower social support.

#### 2.3.2. Work-related social support

Work-related social support was measured by the Swedish version of the Karasek Job Demand Control and Support (JDCS) model (Karasek and Theorell, 1990). This model is commonly used in occupational research and has been found to have good reliability in the Swedish population (Theorell et al., 1988). Participants responded to six statements on a scale from 1 to 4, with high scores indicating low work-related support. After totaling the six scores, we classified police officers scoring above the mean (10.3 ± 2.8) as having low levels of work-related social support.

#### 2.3.3. General mental health

To assess general mental health, we used the 12-item General Health Questionnaire (GHQ-12), a self-administered screening test developed to detect psychiatric disorders in community settings and non-psychiatric clinical settings (Banks et al., 1980; Goldberg and Williams, 1988; Arnetz et al., 2013). The psychometric properties of GHQ-12 have been extensively investigated. Applying the original scoring devised by Goldberg and Williams (1988), the response categories “not at all” and “more than usual” were scored as 0, and the response categories “rather more than usual” and “much more than usual” were scored as 1. The total score thus ranges from 0 to 12, with higher scores indicating poorer mental health.

In accordance with Goldberg’s et al. (1998) recommendation, if the mean is below 1.85 then the threshold of ½ (higher than 2), from 1.85 to 2.7 a threshold of 2/3 (higher than 3), and above 2.7 a threshold of 3/4 (higher than 4) seems to work best for the GHQ-12. In our study, the mean GHQ score was 1.55; thus, we used the cut-off of 2 for the differentiation between individuals with poorer and better mental health.

### 2.4. Statistical analysis

Univariate descriptive statistics were used to describe the sample. Bivariate analyses (*t*-test and chi square) identified continuous and categorical variables (respectively) statistically significantly associated with sex and social support (AVSI, AVAT, ADSI, and ADAT). Pearson correlation coefficient was used to assess the association between GHQ and different types of social support.

The analysis was stratified by sex given the potential differences between women and men regarding sociodemographic variables. Logistic regression modeling calculated crude and adjusted odds ratios (OR) with 95% confidence intervals (CI) and assessed the relationships between different types of social support, sociodemographic variables, and the outcome variable (GHQ). Specifically, univariate logistic regressions were used to calculate crude odds ratios, and multivariate logistic regressions were used to calculate adjusted odds ratios while considering all variables simultaneously and analyzed changes in the magnitude of associations before and after controlling for sociodemographic variables. The model contained 12 independent variables: marital status, children, children aged <18, higher education, work experience, work schedule, main service, social support (AVSI, AVAT, ADSI, and ADAT), and work-related social support. Model fitness was tested by Hosmer – Lemeshow test.

Statistical analyses were performed using SPSS version 28. The significance level was set at *p* < 0.05.

## 3. Results

### 3.1. Univariate statistics and population description

The sample comprised 223 female police officers (30.6%) and 505 male police officers (69.4%). The mean age was 42 years (± 11), with age ranging from 24 to 67 years. Participants’ mean work experience was 15.3 years (±13.2); 59% were working in external service, and 83% were married or cohabitating.

### 3.2. Bivariate analyses

Table 1 shows that mean age and mean years of work experience were higher for male police officers than for female officers. Moreover, higher proportions of male police officers worked in external service, were married/cohabiting, and had children. There were significant differences in social support availability between female and male officers, with the former reporting higher levels of AVSI (4.2 ± 1.5 vs. 3.8 ± 1.5) and AVAT (4.4 ± 1.0 vs. 4.1 ± 1.1). However, female officers also reported lower levels of work-related social support (borderline significance). There was no significant difference in general mental health between male and female police officers. The bivariate associations between sociodemographic and job characteristics, social support, and general mental health by gender is presented in Table 1.

**Table 1:** Sociodemographic characteristics for male (*n* = 505) and female (*n* = 223) police officers.

	Male	Female	Total	*p*-value^a^	Effect size
Age	43.01 (±11.42)	39.28 (±10.51)	41.88 (±11.27)	<0.001	0.023
Age range (years)	24–67	24–65	24–67		
Main service					
External	59.90%	56.50%	58.90%	0.39	0.032
Internal	40.10%	43.50%	41.10%		
Marital status					
Married/cohabiting	86.20%	75.00%	82.80%	<0.001	0.137
Single	13.80%	25.00%	17.20%		
Children					
Yes	79.90%	63.70%	75%	<0.001	0.173
No	20.10%	36.30%	25%		
Children <18 years old					
Yes	54.60%	47.10%	52.30%	0.063	0.069
No	45.40%	52.90%	47.70%		
Higher education (at least bachelor’s degree)					
Yes	83.60%	82.50%	83.30%	0.709	0.014
No	16.40%	17.50%	16.7		
Work experience (years)	16.50 (±13.58)	12.33 (±11.75)	15.25 (±13.17)	<0.001	0.021
Work schedule					
Shift	50.10%	52.70%	50.90%	0.52	0.024
Daytime	49.90%	47.30%	49.10%		
AVSI	3.80 (±1.48)	4.23 (±1.48)	3.93 (±1.49)	<0.001	0.016
AVAT	4.07 (±1.11)	4.43 (±0.96)	4.18 (±1.08)	<0.001	0.023
ADSI	5.80 (±1.83)	5.95 (±1.82)	5.85 (±1.82)	0.298	0.002
ADAT	7.61 (±1.79)	7.32 (±1.89)	7.51 (±1.83)	0.104	0.006
Work-related social support	10.20 (±2.80)	10.65 (±2.96)	10.34 (±2.84)	0.053	0.005
GHQ-12	1.48 (±2.37)	1.74 (±2.50)	1.55 (±2.40)	0.181	0.002

Continuous variables are presented with mean ± 1 standard deviation. AVSI (availability of social integration), AVAT (availability of attachment), ADSI (adequacy of social integration), ADAT (adequacy of attachment), and GHQ-12 (general health questionnaire 12 items). ^a^*p*-value is obtained by *t*-test for continuous variables and chi-square for categorical. Effect size is reported by Cramers’s V for the chi-square analysis and partial et-squared for the *t*-tests.

Table 2 shows the differences between police officers reporting high and low AVSI. There is a significant difference between the percentages of female and male officers reporting high AVSI (68% vs. 56%). Further, 70% of police officers with higher education report high AVSI, compared to 58% of officers without. Table 2 also shows that 64% with better general mental health reported high AVSI, compared to 51% with poorer general mental health.

**Table 2:** Distribution (%) of sociodemographic variables by low and high AVSI (dichotomized by mean value).

Characteristics	AVSI low	AVSI high	*p*-value^a^	Effect size
Age (vs. mean)				
Younger	37.20%	62.80%	0.09	0.066
Older	43.60%	56.40%		
Sex				
Male	43.90%	56.10%	0.003	0.114
Female	31.80%	68.20%		
Marital status				
Married/cohabiting	38.60%	31.40%	0.135	0.059
Single	46.30%	33.70%		
Children				
Yes	41.30%	58.70%	0.451	0.031
No	37.80%	62.20%		
Children <18 years old				
Yes	40.70%	59.30%	0.898	0.008
No	39.90%	60.10%		
Higher education (at least bachelor’s degree)				
Yes	30.30%	69.70%	0.019	0.091
No	42.20%	57.80%		
Work experience (vs. mean)				
Junior	39.10%	60.90%	0.461	0.414
Senior	42.20%	58.80%		
Work schedule				
Shift	43.10%	56.90%	0.105	0.063
Daytime	36.90%	63.10%		
Main service				
Internal	38.60%	61.40%	0.501	0.028
External	41.40%	58.60%		
GHQ-12				
Better mental health	36.30%	63.70%	0.002	0.12
Poorer mental health	49.30%	50.70%		

^a^Chi-square test. AVSI (availability of social integration), GHQ-12 (general health questionnaire 12 items). Effect size is reported by Cramers’s V for the chi-square analysis.

Table 3 shows that 59% of younger police officers reported high AVAT, compared to 40% of older officers. A similar pattern was found regarding work experience, with high AVAT more frequent for officers with fewer years on duty. Like for AVSI, a significantly higher percentage of female officers reported high AVAT (66%, vs. 44% for male officers). There was also a significant difference in AVAT between officers with children (47% high AVAT) and those without (60% high AVAT). In addition, 50% shift workers reported high AVAT, compared to 46% of daytime workers.

**Table 3:** Distribution (%) of sociodemographic variables by low and high AVAT (dichotomized by mean value).

Characteristics	AVAT low	AVAT high	*p*-value^a^	Effect size
Age (vs. mean)				
Younger	41%	59%	<0.001	0.187
Older	59.70%	40.30%		
Sex				
Male	56.20%	43.80%	<0.001	0.2
Female	34.40%	65.60%		
Marital status				
Married/cohabiting	49.00%	51%	0.657	0.021
Single	51.80%	48.20%		
Children				
Yes	52.70%	47.30%	0.004	0.113
No	39.50%	60.50%		
Children <18 years old				
Yes	49.60%	50.40%	0.951	0.005
No	49.10%	50.90%		
Higher education (at least bachelor’s degree)				
Yes	41.50%	58.50%	0.072	0.073
No	51.10%	48.90%		
Work experience (vs. mean)				
Junior	43.80%	56.20%	<0.001	0.161
Senior	60.70%	39.30%		
Work schedule				
Shift	49.60%	50.40%	0.048	0.078
Daytime	53.60%	46.40%		
Main service				
Internal	50.90%	49.10%	0.552	0.026
External	48.30%	51.70%		
GHQ-12				
Better mental health	49.30%	50.70%	0.837	0.011
Poorer mental health	50.50%	49.50%		

^a^Chi-square test. AVAT (availability of attachment), GHQ-12 (general health questionnaire 12 items). Effect size is reported by Cramers’s V for the chi-square analysis.

Table 4 shows that 76% of older police officers reported high ADSI, compared to 67% of younger police officers. A similar pattern was found regarding work experience, with high ADSI more frequent for officers with more years on duty. Table 4 also shows that 78% of officers with better general mental health reported high ADSI, compared to 55% of those with poorer general mental health. Table 5 shows that 66% of police officers with better general mental health reported high ADAT, compared to 53% of those with poorer general mental health.

**Table 4:** Distribution (%) of sociodemographic variables by low and high ADSI (dichotomized by mean value).

Characteristics	ADSI low	ADSI high	*p*-value^a^	Effect size
Age (vs. mean)				
Younger	32.50%	67.50%	0.013	0.099
Older	23.50%	76.50%		
Sex				
Male	29.80%	70.20%	0.31	0.042
Female	25.70%	74.30%		
Marital status				
Married/cohabiting	26.90%	73.10%	0.096	0.068
Single	35.00%	65.00%		
Children				
Yes	27.50%	72.50%	0.371	0.038
No	31.40%	68.60%		
Children <18 years old				
Yes	31.10%	68.90%	0.098	0.067
No	25.10%	75.90%		
Higher education (at least bachelor’s degree)				
Yes	25.60%	74.40%	0.578	0.026
No	28.70%	71.30%		
Work experience (vs. mean)				
Junior	32.20%	67.80%	0.002	0.122
Senior	20.60%	79.40%		
Work schedule				
Shift	34.10%	65.90%	<0.001	0.129
Daytime	22.40%	77.60%		
Main service				
Internal	27.30%	72.70%	0.595	0.024
External	29.50%	70.50%		
GHQ-12				
Better mental health	22.20%	77.80%	<0.001	0.233
Poorer mental health	45.50%	54.50%		

^a^Chi-square test. ADSI (adequacy of social integration), GHQ-12 (general health questionnaire 12 items). Effect size is reported by Cramers’s V for the chi-square analysis.

**Table 5:** Distribution (%) of sociodemographic variables by low and high ADAT (dichotomized by mean value).

Characteristics	ADAT low	ADAT high	*p*-value^a^	Effect size
Age (vs. mean)				
Younger	37.70%	62.30%	0.733	0.02
Older	35.70%	64.30%		
Sex				
Male	34.10%	65.90%	0.112	0.077
Female	41.90%	58.10%		
Marital status				
Married/cohabiting	35.30%	64.70%	0.253	0.058
Single	42.70%	57.30%		
Children				
Yes	38.00%	62.00%	0.343	0.048
No	32.80%	67.20%		
Children <18 years old				
Yes	40.70%	59.30%	0.059	0.091
No	31.90%	68.10%		
Higher education (at least bachelor’s degree)				
Yes	28.90%	71.10%	0.131	0.075
No	38.10%	61.90%		
Work experience (vs. mean)				
Junior	37.30%	62.70%	0.638	0.026
Senior	34.60%	65.40%		
Work schedule				
Shift	36.00%	64.00%	0.722	0.021
Daytime	38.00%	62.00%		
Main service				
Internal	40.60%	59.40%	0.18	0.066
External	34.20%	65.80%		
GHQ-12				
Better mental health	33.80%	66.20%	0.01	0.125
Poorer mental health	47.50%	52.50%		

^a^Chi-square test. ADAT (adequacy of attachment), GHQ-12 (general health questionnaire 12 items). Effect size is reported by Cramers’s V for the chi-square analysis.

### 3.3. Pearson correlation coefficient

Table 6 shows the findings of Pearson correlation coefficient. The results show significant correlations between general mental health and all different types of social support variables for both females and males, except for general mental health and AVSI for females, and general mental health and ADSI for males. In addition, the results show correlations between all different types of social support for females and males, except between AVSI and work-related social support for females. Low social support is correlated with worse general mental health.

**Table 6:** Pearson correlations between AVSI^1^, AVAT^1^, ADS^1^, ADAT^1^, work-related social support^2^, and GHQ^3^ by gender.

	GHQ-12	AVSI	AVAT	ADSI	ADAT	Work-related social support
GHQ-12	1	−0.104	−0.200**	−0.329**	−0.370**	0.299**
	1	−0.166**	−0.101*	−0.231	−0.168**	0.302**
AVSI		1	0.389**	0.407**	0.331**	−0.11
		1	0.268**	0.349**	0.209**	−0.174**
AVAT			1	0.376**	0.549**	−0.188**
			1	0.236**	0.275**	−0.194**
ADSI				1	0.571**	−0.177**
				1	0.564**	−0.176**
ADAT					1	−0.221**
					1	−0.152**
Work-related social support						1
						1

*p < 0.05; **p < 0.01 (1st row: female, 2nd row: male), ^1^ low score is low support, ^2^ high score is low support, ^3^ high score is worse mental health. AVSI (availability of social integration), AVAT (availability of attachment), ADSI (adequacy of social integration), ADAT (adequacy of attachment), GHQ-12 (general health questionnaire 12 items).

### 3.4. Univariate logistic regressions

Table 7 shows the findings of univariate logistic regressions on poorer general mental health. The results show that the odds of reporting poorer mental health were 2.7 times higher for male police officers with low work-related social support, compared to those with high work-related social support (odds ratio (OR) = 2.66, 95% confidence interval (CI): 1.76–4.01, *p* < 0.001). A similar result was obtained for female officers (OR = 2.42, 95% CI: 1.35–4.36, *p* = 0.003). The odds of reporting poorer mental health were 1.8 times higher for single men compared to married men (OR = 1.86, 95% CI: 1.09–3.19, *p =* 0.023), and two times higher for single women than for married women (OR = 2.00, 95% CI: 1.07–3.74, *p* = 0.031). Male police officers with low AVSI were 1.8 times more likely to report poorer mental health compared to those with high AVSI (OR = 1.80, 95% CI: 1.20–2.69, *p* = 0.004). Likewise, male officers with low ADSI were 2.9 times more likely to report poorer mental health compared to those with high ADSI (OR = 2.86, 95% CI: 1.85–4.43, *p* < 0.001). Female officers with low ADSI were also three times more likely to report poorer mental health compared to those with high ADSI (OR = 3.22, 95% CI: 1.70–6.14, *p* < 0.001).

**Table 7:** Estimated odds ratios (OR 95% CI) for poorer mental health by different categories of social support and sociodemographic variables among male and female police officers.

	Male		Female	
Predictors	Crude OR (95% CI)	Adjusted OR (95% CI)	Crude OR (95% CI)	Adjusted OR (95% CI)
Marital status Married/cohabiting (ref) Single	1.86 (1.09–3.19)*	3.25 (1.33–7.94)*	2.00 (1.07–3.74)*	1.22 (0.46–3.23)
Children Yes (ref) No	1.21 (0.74–1.96)		1.36 (0.76–2.42)	
Children <18 years old Yes (ref) No	1.07 (0.72–1.59)	0.88 (0.43–1.78)	1.20 (0.69–2.12)	1.63 (0.69–3.85)
Higher education (at least bachelor’s degree) Yes (ref) No	0.71 (0.43–1.18)	0.79 (0.37–1.72)	1.42 (0.64–3.12)	1.30 (0.41–4.10)
Work experience (vs. mean) Junior (ref) Senior	0.66 (0.43–1.00)	1.17 (0.48–2.89)	0.72 (0.37–1.40)	0.56 (0.16–1.93)
Work schedule Daytime (ref) Shift work	1.87 (1.25–2.80)**	1.56 (0.61–3.95)	1.12 (0.63–1.97)	0.63 (0.19–2.10)
Main service Internal (ref) External	1.54 (1.01–2.32)*	1.55 (0.56–4.23)	0.92 (0.52–1.61)	1.29 (0.38–4.43)
AVSI <mean = low (ref)	1.80 (1.20–2.68)**	1.08 (0.56–2.10)	1.59 (0.90–2.83)	1.46 (0.65–3.27)
AVAT <mean = low (ref)	1.02 (0.67–1.55)	0.74 (0.37–1.50)	1.36 (0.74–2.50)	0.64 (0.23–1.76)
ADSI <mean = low (ref)	2.86 (1.85–4.43)***	1.04 (0.45–2.44)	3.22 (1.70–6.14)***	1.11 (0.42–2.95)
ADAT <mean = low (ref)	1.09 (0.62–1.89)	0.95 (0.43–2.08)	3.51 (1.75–7.03)***	2.79 (1.07–7.27)*
Work-related social support <mean = low (ref)	2.66 (1.76–4.01)***	3.55 (1.82–6.94)***	2.42 (1.35–4.36)**	1.56 (0.70–3.48)

*p < 0.05 **p < 0.01 ***p < 0.001. AVSI (availability of social integration), AVAT (availability of attachment), ADSI (adequacy of social integration), ADAT (adequacy of attachment), GHQ-12 (general health questionnaire 12 items), CI (confidence interval).

Male police officers in external service were 1.5 times more likely to report poorer mental health compared to those in internal service (OR = 1.54, 95% CI: 1.01–2.32, *p* = 0.043). Moreover, male officers who worked shifts were 1.9 times more likely to report poorer mental health compared to those who worked daytime hours (OR = 1.87, 95% CI: 1.45–2.80, *p* = 0.002). Finally, the risk of reporting poorer mental health was 3.5 times higher for female officers with low ADAT than for those with high ADAT (OR = 3.51, 95% CI: 1.75–7.03, *p* < 0.001).

### 3.5. Multivariate logistic regressions

Table 7 also reports the results of the multivariate logistic regressions. Age was excluded because of its high correlation with work experience. The results show that the risk of reporting poorer mental health was over 3.5 times higher for male police officers with low (vs. high) work-related social support (OR = 3.55, 95% CI: 1.82–6.94, *p* < 0.001). Single men were 3.2 times more likely to report poorer mental health compared to married men (OR = 3.25, 95% CI: 1.33–7.94, *p* = 0.01). Finally, female police officers with low ADAT were nearly three times more likely to report poorer mental compared to those with high ADAT (OR = 2.79, 95% CI: 1.07–7.27, *p* = 0.036). Hosmer-Lemeshow test showe non-significant chi-square test which indicates that the data fitted the model for both men and women.

## 4. Discussion

Focusing on Swedish police officers, this paper aimed to analyze different types of social support, including availability and adequacy of social support in relation to general mental health. The first objective of this study was to specifically investigate, availability and adequacy of aspects of different types of social support (social integration and attachment) in relation to general mental health. The second objective was to assess sex difference in term of the associations between different types of social support. The third objective was to assess the role of sociodemographic variables between different types of social support.

The main findings show a noteworthy relationship between social support availability and sex, with female police officers reporting higher levels of both availability of social interaction and availability of attachment compared to male officers. However, we found no difference between the sexes regarding adequacy of social support. These results are somewhat consistent with prior findings that women perceive higher levels of social support compared to men, although many studies do not differentiate quantity and quality in social support (Rueger et al., 2008, 2010). However, Kaur et al. (2021) found that men relied more on families or spouses whereas women sought more support from friends or relationships based on reciprocity. There are also studies showing no differences between men and women regarding social support (Barnett et al., 2020). In addition, Verhofstadt et al. (2007) showed that self-report measures yielded significant gender differences in support soliciting and provision whereas observational measures did not. Thus, social support can be available although not perceived as adequate. A future investigation of gender differences in the quantity and quality of social support can highlight those relationships that are most important. The results of the current study illustrate the need to be aware of these distinctions when comparing results across social support studies.

Availability of social interaction was higher for police officers with higher education than for those without. Police officers with better general mental health also reported higher availability of social interaction, adequacy of social interaction, and adequacy of attachment compared to those with poorer mental health. The associations between adequacy of social interaction and adequacy of attachment are somewhat consistent with prior findings on the role of social support in post-traumatic growth (Scrignaro et al., 2011). Scrignaro et al. (2011) suggest that cancer patients who received social support that satisfied their psychological needs experienced post-traumatic growth in the short term, but no such association in the long term. However, the relationships between social support and general positive outcomes are clearly recognized in the literature (Bonanno et al., 2007; Shnaider et al., 2017; Arble et al., 2018). Notably, the associations between qualitative social support (adequacy of social interaction and adequacy of attachment) and general mental health are consistent with the findings of Nero et al. (2022), who reported that higher perceived quality of social support was associated with reduced levels of mental health symptoms.

Furthermore, police officers who worked shifts, were younger, had less work experience, and/or had no children reported higher availability of attachment. By contrast, older police officers reported higher adequacy of social interaction compared to younger officers, and a similar pattern was found regarding work experience. A possible explanation might be that younger officers have a big circle of friends whereas over time working as a police officer generates quality in social interactions. Another possible explanation might be the suggestion by Henderson et al. (1980) that requirements of social support diminish with age, meaning that older police officers are more tolerant than younger officers of having social relationships. However, this is somewhat contradicted by Nisbet et al. (2023), who reported that cadets had significantly higher perceived social support than serving officers in the Royal Canadian Mounted Police, based on both item scores and the overall total for Social Provisions Scale (SPS-10).

In our study, police officers who were male, single, and/or had low work-related social support reported poorer mental health. A plausible explanation might be that single male police officers are strongly committed to their job and, without sufficient work-related social support, this commitment has a negative effect on their mental health. Married police officers might obtain necessary distance from their job through their spouse. This is confirmed by Nisbet et al. (2023), who reported that cadets who were married or in a common-law relationship had the highest levels of perceived social support, which in turn reduced the odds of screening positive for generalized anxiety disorder, social anxiety disorder, and panic disorder. However, Nisbet et al. (2023) reported no gender differences in the perceived level of social support. Future studies could include overtime work and workload in relation to marital status and social support to analyze and explore how those factors are associated to general mental health.

Female police officers with low adequacy of attachment reported poorer mental health than those with high adequacy of attachment. A possible explanation might be that less satisfaction with perceived social support is associated with poorer mental health but having few close relationships does not negatively affect a person’s perceived mental health (Henderson et al., 1980; Prati and Pietrantoni, 2010). Another explanation might be that the hegemonic masculinity culture within the police force (Andersson, 2003; Myers et al., 2004; Dahlgren, 2007) causes female officers without close qualitative relationships to experience poorer mental health.

### 4.1. Limitations

It should be noted that the current study was limited in several ways. First, we used self-report measures for both predictor and outcome variables. This may have introduced biases, for instance resulting from respondents’ misunderstanding and misinterpretation. While self-report of perceived social support is essential by nature, observational measures should also be used. Second, using a convenience sample of police officers might have affected the results. Nevertheless, as this is not a descriptive prevalence study, the bias may be only a minor problem. Third, this study used a cross-sectional design, thus preventing inference of causality. However, the findings offer important insights on the associations between different variables such as sex, work experience, shift work, having children, the different dimensions of social support, and general mental health. Finally, the data were collected in 2014 and so might be outdated. Nonetheless, our analyses reveal the associations between social support within and outside work and general mental health among Swedish police officers before the 21 sovereign police authorities were combined to form the National Police Authority. This makes it possible to make comparison between social support and general mental health before and after the Swedish police reorganization to one authority. Furthermore, even if organizational factors and social support can have changed over time this is an association study and therefore it is still applicable in today’s police work.

### 4.2. Implications

The current study has implications for police practice. It is important to address the variation in quantitative and qualitative aspects of perceived social support among different groups. This study also adds to the literature on different dimensions of social support and the associations with general mental health for police officers. The findings from the study by Jetelina et al. (2020) emphasize that stigma is a prevalent and significant barrier to mental health care among police officers. Encouraging a culture where colleagues and supervisors actively support each other’s mental well-being and understand the importance of seeking help can be a critical part of reducing the stigma surrounding mental health care in law enforcement. Violanti (2020) suggests that creating an environment where officers feel supported and encouraged to seek help for their mental health is crucial. This can involve leadership demonstrating their commitment to mental health initiatives. Addressing the stigma surrounding mental health care within the police force is a complex but vital endeavor. Our work takes an important step in improving understanding of differences in perceived social support. Future research should focus on systematically clarifying the important influences of quantitative and qualitative aspects of social support on police officers’ general mental health, considering matters of unique and independent effects in the interpretation of findings, as well as making comparisons across time and between studies.

### 4.3. Conclusion

Based on this study’s findings, police organizations need to be aware of variations in and effects of social support in their efforts to make the work environment more supportive. First, we found that low adequacy of attachment was associated with poorer general mental health among female officers, although female officers reported higher levels of both availability of social integration and availability of attachment compared to male officers. Thus, the quality of close social relationships seems important to female officers’ mental health. Second, we found that low work-related social support was associated with poorer mental health among single male police officers. Police organizations should ensure that this group of officers receives necessary social support at work. Third, police officers who work shifts, are younger, have less work experience, and/or do not have children reported higher levels of availability of attachment, whereas older police officers reported higher adequacy of social integration compared to younger police officers. Social support can buffer the stresses inherent in police work, although the associations are complex. Given the individual differences in qualitative and quantitative social support the police organizations should develop individual tailored peer-support program and address those officers’ demanding work and need for support. A peer-support program might help to improve officers’ psychological resilience and cultivate a help-seeking culture by reducing stigma. Police organizations need to be aware of the different effects of social support inside and outside work on mental health.

## Data availability statement

The raw data supporting the conclusions of this article will be made available by the authors, without undue reservation.

## Ethics statement

The studies involving humans were approved by the Regional Ethical Review Board at Umeå University, Sweden, Diarie number 2014/69-31. The studies were conducted in accordance with the local legislation and institutional requirements. The participants provided their written informed consent to participate in this study. Written informed consent was obtained from the individual(s) for the publication of any potentially identifiable images or data included in this article.

## Author contributions

JH and MP: conceptualization, methodology, and analysis and interpretation of data and writing—(review and editing). MP: validation. JH: data collection and project administration and writing—original draft preparation. All authors contributed to the article and approved the submitted version.
